# Fracture Resistance of Repaired 5Y-PSZ Zirconia Crowns after Endodontic Access

**DOI:** 10.3390/dj11030076

**Published:** 2023-03-07

**Authors:** Andreas Greuling, Mira Wiemken, Christoph Kahra, Hans Jürgen Maier, Michael Eisenburger

**Affiliations:** 1 Department of Prosthetic Dentistry and Biomedical Materials Science, Hannover Medical School, Carl-Neuberg-Straße 1, 30625 Hannover, Germany; 2 Institut für Werkstoffkunde (Materials Science), Leibniz University Hannover, An der Universität 2, 30823 Garbsen, Germany

**Keywords:** fracture load, repaired crowns, zirconia, endodontic access, chewing simulation

## Abstract

This study analyzed the fracture load before and after a chewing simulation of zirconia crowns that were trepanned and repaired using composite resin. Overall, 3 groups with 15 5Y-PSZ crowns in each group were tested. For group A, the fracture load of the unmodified crowns was evaluated. For group B, the crowns were trepanned and repaired using composite resin, also followed by a fracture test. For group C, crowns were prepared like in group B but received thermomechanical cycling before the final fracture tests. Furthermore, scanning electron microscopy (SEM) and X-ray microscopy (XRM) analysis were performed for group C. The mean fracture loads and standard deviation were 2260 N ± 410 N (group A), 1720 N ± 380 N (group B), and 1540 N ± 280 N (group C). Tukey-Kramer multiple comparisons showed a significant difference between groups A and B (*p* < 0.01) and groups A and C (*p* < 0.01). After ageing, surface fissures were detected via SEM, but no cracks that reached from the occlusal to the inner side of the crown were detected via XRM. Within the limitations of this study, it can be stated that trepanned and composite-repaired 5Y-PSZ crowns show lower fracture loads than 5Y-PSZ crowns without trepanation.

## 1. Introduction

The preservation of natural teeth is one of the main goals of dentistry. When defects caused by caries become too large, teeth are often restored using crowns. Nowadays, all-ceramic zirconia crowns are widely used for this purpose, as they lead to an aesthetic and robust restoration.

Zirconia-based materials, often with yttria as a phase stabilizer, are known for excellent mechanical properties and have been well-established in dentistry [1]. The main reason for their high fracture strength lies in the martensitic phase transformation, which has been explained in detail in the work by Kelly et al. [2]. In the past decades, zirconia-based ceramics were used successfully to fabricate fixed dental prostheses, including dental crowns [3]. Especially first generation zirconia was often used with a veneering, which resulted in improved esthetics [4]. Unfortunately, the veneering may lead to problems like chipping, and its mechanical properties are inferior to monolithic zirconia [5,6,7]. Over time zirconia-based materials with higher amounts of yttria, like 5Y-PSZ, became more popular as they provide better optical properties than monolithic 3Y-TZP while having a lower but still sufficient strength [4,8].

Sometimes, a tooth with a crown needs an endodontic treatment after some time. One common solution is to remove the crown, perform the endodontic treatment and place a new crown onto the abutment tooth [9,10]. An alternative, which is sometimes chosen in praxi when the crown margin is still sufficient, is to trepan directly through the crown, perform the endodontic treatment and repair the crown afterwards using composite [11]. If successful, this approach saves time and cost for the patient.

The bond strength of resin-based materials to acid-resistant ceramics like Y-TZP or Y-PSZ has been reported as not durable in several studies in the past for some material combinations [12,13,14]. However, suitable surface conditioning such as air particle abrasion enables a durable bond between composite and zirconia. Wolfart et al. [15] for instance recommended MDP-containing composite on air-abraded zirconia ceramic as a promising bonding method. Cristoforides et al. [16] compared different repair methods after ageing and found a tribochemical silica coating process (TBS) to be superior compared to MDP-containing liners. Oyagüe et al. [17] have found air particle abrasion to have a positive effect on the bond strength of zirconia ceramics to dual-cured resin cements. Xible et al. [18] found that TBS and following silanization increased the bond strength between zirconia-based ceramics and composite resin. When combined with particle blasting the universal MDP-containing adhesive Clearfil Ceramic primer plus (Kuraray Europe, Main, Germany), which is used in this study, has been found to yield a good bond strength between zirconia and composite resin [19].

Furthermore, Oyagüe et al. [17] found that water aging plays an important role in zirconia-to-composite chemical bonds. The water environment also plays a role in the fracture mechanics of zirconia, as the fracture toughness of zirconia is significantly lower in water than in air [20]. Besides water aging, mechanical aging is also an important factor and chewing simulations are often performed in the field of dentistry for mechanical aging [21,22,23]. (Thermo-) mechanical cycling is known to decrease the remaining fracture strength of ceramic crowns in many cases [24]. Besides fracture strength, marginal accuracy and microbiological leakage are also a concern when crowns are tested for clinical usage [25].

The current study aimed to study how the fracture resistance of trepanned zirconia crowns that were repaired after endodontic access behaves in comparison to zirconia crowns without trepanation. The null hypothesis was that there is no difference between the tested groups.

## 2. Materials and Methods

**Figure 1:**
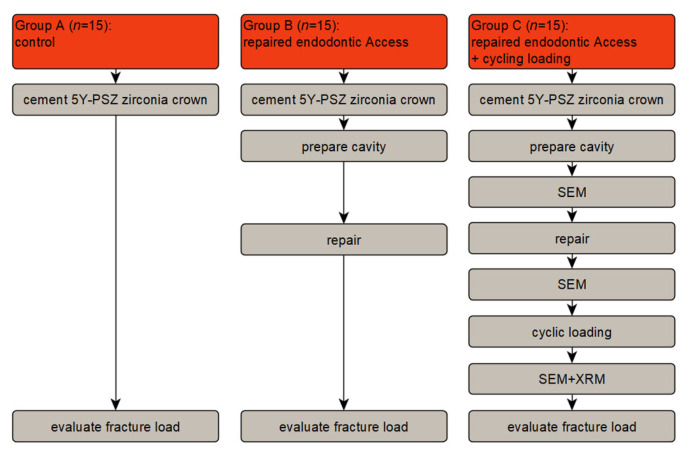
Schematic overview of the study design: Scanning Electron Microscopy (SEM) imaging was done for 5 specimens, whereas characteristic results are shown exemplarily; X-Ray Microscopy (XRM) analysis was performed for one specimen. 5Y-PSZ: Partially Stabilized Zirconia with 5 mol% Yttria.

In this study the fracture load of 5Y-PSZ zirconia crowns made from Z-CAD^®^ smile (Metoxit, Thayngen, Switzerland), which were prepared for endodontic access and repaired afterwards, was determined. A schematic overview of the study design is shown in Figure 1. For group A (control), 5Y-PSZ zirconia crowns cemented on polyurethane model stumps were loaded to fracture. For group B, the zirconia crowns were prepared with a cavity for endodontic access, repaired and then loaded to fracture. Group C is the same as group B, but with an additional chewing simulation before the final loading to fracture. Details of the procedures are described below.

### 2.1. Specimen Preparation

#### 2.1.1. Abutment Teeth

In order to create model abutment teeth, a model tooth 16 (KaVo, Biberach, Germany) was prepared for a full-ceramic crown using diamond burs (TD3355, Komet Dental, Lemgo, Germany). The tooth was reduced by 2 mm occlusally and a circular chamfer preparation with a depth of 1 mm and an angle of 7° was prepared. Edges were smoothed to reduce later stress peaks. A silicone replica (Flexitime Easy Putty, Kulzer, Hanau, Germany), made before the preparation, was used to check the substance removal.

Afterwards, a plaster cast of the model abutment tooth was digitized with a dental scanner (Ceramill map 400, Amann Girrbach, Pforzheim, Germany) and modified using Meshmixer (Autodesk, San Rafael, USA) and Rhinoceros 7.0 (McNeel North America, Seattle, WA, USA). A frustum of a cone was added 1 mm below the preparation margin, whereas the base of the frustum had a diameter of 20 mm. Furthermore, a notch was added to the frustum, which was later used for reproducible positioning and to prevent rotation.

**Figure 2:**
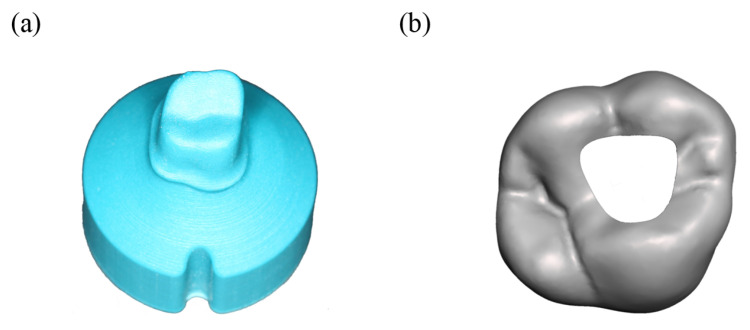
(**a**) abutment tooth used for crown support. (**b**) 3d-model of tooth 16 in occlusal view, with a cavity for endodontic access.

Based on the digital model described before a negative form was created via CAD and 3D-printed with a Form 2 printer (Formlabs, Berlin, Germany) and Dental SG (Formlabs, Berlin, Germany). Finally, this negative form was used to create polyurethane model abutment teeth, using AlphaDie MF (Schütz-Dental, Rosbach vor der Höhe, Germany). The finished abutment teeth (see Figure 2a) were blasted with Al_2_O_3_ particles (50 µm) at 1 bar.

#### 2.1.2. Crown Production

Using the program “Ceramill” (Amann Girrbach, Pforzheim, Germany) a crown for the polyurethane model abutment teeth was designed, with a minimum thickness of 0.7 mm and a cement gap of 0.06 mm. The crown was exported as an STL-File. Using a Ceramill motion 2 (Amann Girrbach GmbH, Pforzheim, Germany) milling machine, monolithic 5Y-PSZ crowns were milled from Z-CAD^®^ smile (Metoxit, Thayngen, Switzerland) round blanks. After milling, the crowns were carefully detached from the blank by a professional dental technician. Afterwards, the crowns were sintered according to the manufacturer’s instructions. The sintered crowns were coated with two layers of IPS e.max Ceram Glaze Paste FLUO, (Ivoclar Vivadent GmbH, Ellwangen, Germany) and sintered at 850 °C as specified by the manufacturer. Finally, the crown lumen was blasted with Al_2_O_3_ particles (50 µm) at 2 bar.

#### 2.1.3. Crown Cementation

The monolithic zirconia crowns were cemented onto the polyurethane abutment teeth using a glass ionomer cement (3M Ketac cem Aplicap, 3M Deutschland, Neuss, Germany). The cement was prepared according to the manufacturer’s instructions and applied to the crown, which was then manually pressed onto the abutment tooth for 10 s at a nominal force of 25 N. During the process, a weighing scale measured the force. Excess cement was removed.

#### 2.1.4. Cavity for Endodontic Access

In order to simulate a cavity suitable for endodontic access, a cavity was created in the crowns STL file mentioned earlier (see Figure 2b). This file was then used to create a drilling template. Using this template, an experimental setup using a milling unit (Ceramill, Amann Girrbach, Pforzheim, Germany) and a laboratory turbine (Presto Aqua, Amann Girrbach, Pforzheim, Germany) was constructed. This setup allowed for a cavity with a reproducible geometry. The crown was trepaned under water-cooling using a dental bur ZR6881.314.016 (Komet Dental, Lemgo, Germany) with manual pressure. After penetrating the crown, the grinding depth of the bur into the polyurethane was <0.5 mm.

#### 2.1.5. Repairing the Crown and Polishing

The access cavities were repaired using a universal adhesive (Clearfil Ceramic primer plus, Kuraray Europe, Main, Germany) and a composite (Estelite sigma Quick, Tokuyama Dental Deutschland, Altenberge, Germany). The universal adhesive was applied for 20 s, followed by up to 10 s removal using compressed air. Afterwards, the cavity was filled with composite resin in two increments and treated with a polymerisation lamp (Bluephase G2, Ivoclar Vivadent, Ellwangen, Germany) for 20 s in each step. After that, the composite filling in the crown and the crowns surface close to the repaired section were polished using coarse, fine and very fine polishing discs (2381C, 2381F, 2381SF, Sof-Lex XT discs, 3M, Neuss, Germany) at rotation speeds of 10.000–15.000 rpm. Furthermore, polishing discs EVE Diacomp Plus Twist-Set (EVE, Keltern, Germany) were used afterwards at a rotation speed of 6.000 rpm. All polishing was performed using water cooling.

### 2.2. Thermomechanical Testing

The specimens of group C were aged using thermomechanical cycling in a chewing simulator (Chewing Simulator CS-4.8, SD Mechatronik, Feldkirchen-Westerham, Germany). An axial force of 90 N was applied using a spherical steatite antagonist with a diameter of 6 mm. The mechanical cycling was performed at about 1.2 Hz for 10^6^ cycles, meant to model about 2 years of ageing. Furthermore, about 6000 cycles of thermocycling were applied parallel to mechanical cycling, whereas water at 5 °C and 55 °C was pumped to the test chambers. Furthermore, a water additive (ThermoClean DC, Bioanalytic, Waldmatten, Germany) was used, which reduces foaming and bacteria.

### 2.3. Fracture Test

The fracture load for specimens from all groups was finally tested in a universal testing machine (UTS 20 kN modernized, Zwick GmbH & Co. KG, Ulm, Germany). Before testing, all specimens were stored in water at 36 °C for 24 h. A uniaxial force was applied using a 6 mm stainless steel sphere. The sphere was placed at the side of the mesiopalatal, mesiobuccal and distobuccal cusp and occlusion foil (Arti-Fol, 8 µ rot, Dr. Jean Bausch, Köln, Germany) was used to document the resulting 3-point contact details. The force was increased using a constant crosshead displacement rate of 0.5 mm/min until fracture. Fracture detection was programmed as a force drop of 30% below the maximum. The achieved maximum is referred to as the fracture load in the following.

### 2.4. Scanning Electron Microscopy (SEM)

SEM imaging was performed for 5 specimens of group C after the cavity was prepared, after it was repaired and after the chewing simulation (Figure 1). For clarity, the result section shows only a characteristic selection of those images. Before imaging, the specimens were vacuum dried at room temperature. Imaging was done with a Zeiss EVO MA 10 (Carl Zeiss, Oberkochen, Germany) without sputtering at pressures of roughly $5\times 10^{-4}$ Pa using a Secondary Electron Detector operating at variable pressure (VPSE).

### 2.5. X-ray Microscopy (XRM)

After chewing simulation, an XRM measurement was carried out for one specimen of group C. Using the flat panel detector a tomography was created with a Zeiss Xradia 520 Versa (Carl Zeiss AG, Oberkochen, Germany) X-ray microscope. At an accelerating voltage of 160 keV and an exposure time of 0.16 s, 1601 projections were acquired. The voxel size was 7.65 µm. The software Dragonfly 2020 (ORS, Montréal, QC, Canada) was used to evaluate the tomography and to create the images.

### 2.6. Statistical Analysis

Statistical analysis was performed using Matlab R2022a (The Mathworks). Tests for normal distribution (Kolmogorov-Smirnow), a one-way anova and posthoc multiple comparisons (Tukey-Kramer) were computed. The analysis was done on a significance level of 0.05.

## 3. Results

**Figure 3:**
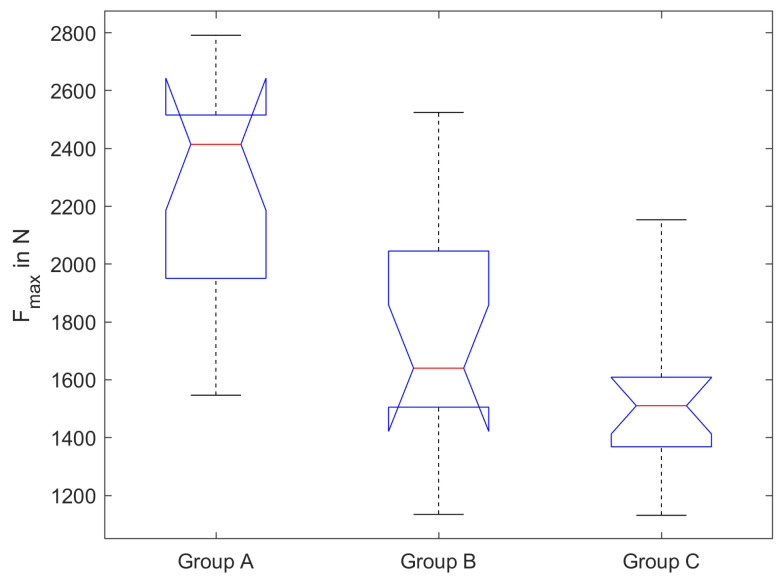
Fracture load for crowns from group A (control), group B (trepanned and repaired without ageing) and group C (trepanned and repaired with ageing), shown in a notched boxplot; the red lines mark the median and the notches mark the 95% confidence interval; *n* = 15 for each group.

The results of the fracture test are shown in Figure 3. The mean fracture loads and standard deviation are 2260 N ± 410 N (group A), 1720 N ± 380 N (group B) and 1540 N ± 280 N (group C). A Kolmogorov-Smirnov test failed to reject the hypothesis that the results for groups A, B and C were drawn from a population with normal distribution. To put it simply, normal distribution of the underlying population is likely according to the performed test. A one-way anova showed a significant difference in the fracture load between the three groups (*p* < 0.05). Tukey-Kramer multiple comparisons show a significant difference between groups A and B (*p* < 0.01) and groups A and C (*p* < 0.01). Between groups B and C, no significant difference can be found (*p* = 0.37).

**Figure 4:**
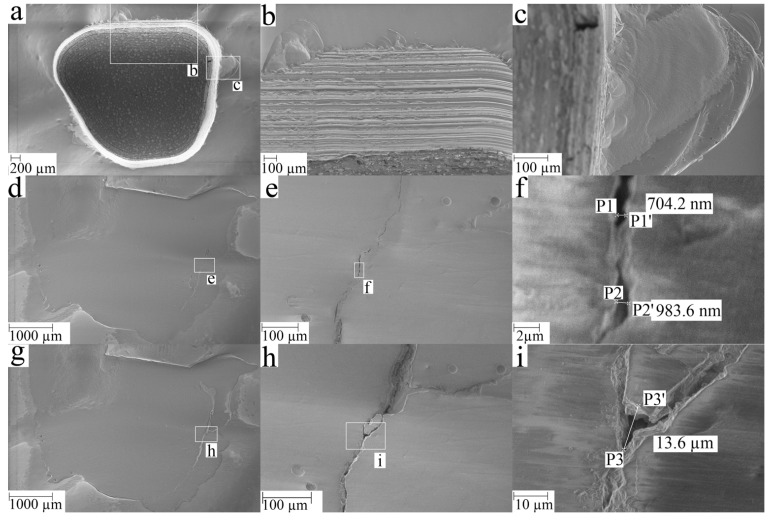
Exemplary SEM-image of a trepanned crown at various stages in the process (all images for the same specimen from group C: (**a**) overview of the trepanned crown without repair, (**b**) enlarged section on the oral side showing grinding grooves and chipping at the occlusal surface, (**c**) enlarged defect on occlusal surface, (**d**) overview of the repaired area (before CS), (**e**) enlarged section of the repaired area (before CS), (**f**) further enlarged section of the repaired area (before CS), (**g**) overview of the repaired area (after CS), (**h**) enlarged section of the repaired area (after CS), (**i**) further enlarged section of the repaired area (after CS). The points P1, P1’ (same applies for P2, P2’, P3, P3’) are start and end point of the measured distance.

SEM images of one crown from group C are shown in Figure 4. After crown trepanation, some chipping can be observed on the occlusal surface next to the cavity (Figure 4a–c). The repairing composite overlaps the trepanned area and covers the chipped areas (Figure 4d). Before the chewing simulation, ditches are visible in Figure 4d–f, which in the case of f) had a width of ≈1 µm. After the chewing simulation, some of the material at the margin of the trepanned area disappear and widening of the ditches can be observed (>10 µm in some areas) (Figure 4g–i). These observations are typical and similar observations were made for other specimens.

**Figure 5:**
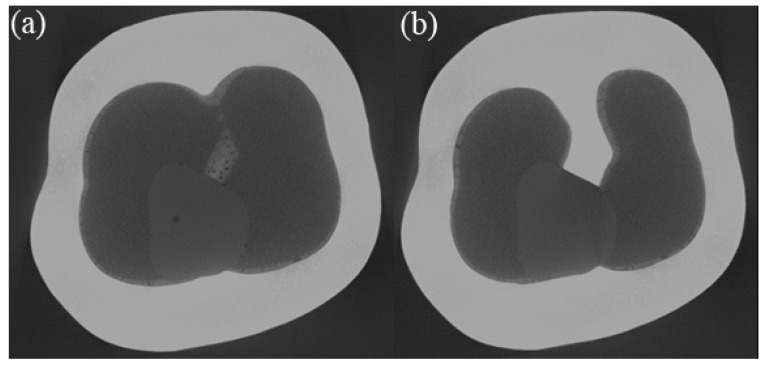
XRM-scans of a repaired crown (specimen C1): (**a**) slice taken close to the cement-crown interface, where pores in the cement, as well as pores in the repairing composite, are visible; (**b**) slice which is about 0.19 mm closer to the occlusal plane than (**a**); nearly no pores are visible at the crown-composite interface.

The depth of the ditches could not be determined as the SEM only captures the near-surface region. In order to evaluate whether cracks propagated from the occlusal to the internal side of the crown an XRM scan was performed. Due to the specimen’s chemical composition and dimensions, the transmission was only about 5%, which resulted in a rather low resolution (voxel edge length of 7.65 µm). Figure 5a) exemplarily shows pores observable at the crown cement interface and pores at the interface of the repairing composite and the crown. However, these pores do not cover the full distance from the internal surface to the occlusal surface (Figure 5b). This observation is limited by the voxel size stated above.

**Figure 6:**
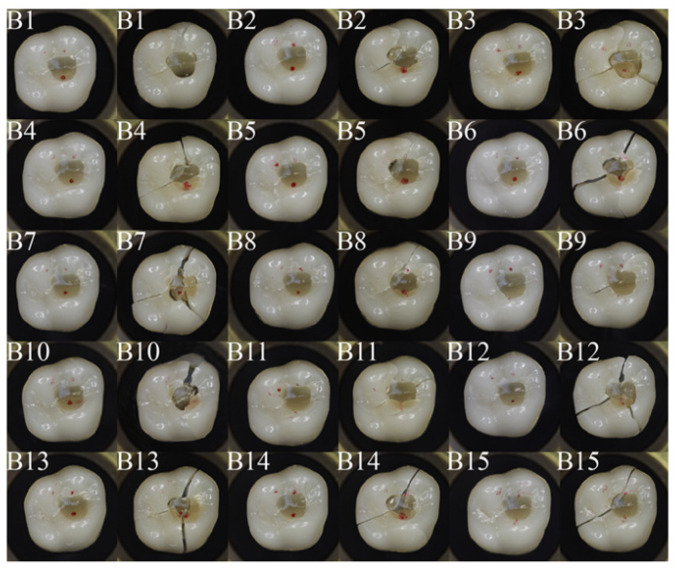
Crowns from group B, before and after the fracture test; the red features indicate the areas where contact with the steel ball occurred; post-fracture the remaining pieces were reconstructed.

**Figure 7:**
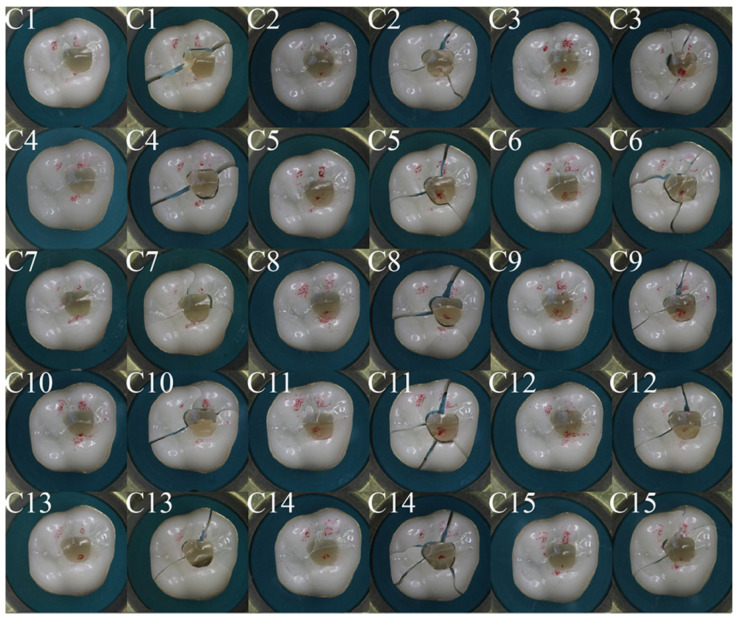
Crowns from group C, before and after the fracture test; the red features indicate the areas where contact with the steel ball occurred; post-fracture the remaining pieces were reconstructed.

Figure 6 and Figure 7 show occlusal views of all crowns tested in group B and group C, with the contact areas between the crown and antagonist sphere marked in red. Variations in the contact situation can be observed. They originate from slight height differences in the manually repaired segments. In group B, 12 specimens had two contacts on the zirconia part of the crown and one contact on the repaired area. Three specimens had two contacts on the zirconia part of the crown and one contact close to the margin of the repaired area. In group C, 10 specimens had two contacts on the zirconia part of the crown and one contact on the repaired area. Five specimens had two contacts on the zirconia part of the crown and one contact close to the margin of the repaired area. If one contact was on the repaired area, the crowns often show a crack through this contact area, whereas the main cracks are oriented from the repair site in distal direction, in mesio-buccal direction, and in mesio-palatal direction. If no contact was on the repaired area, the crack path can be observed from disto-palatal to mesio-buccal. This behavior is similar for groups B and C.

## 4. Discussion

The present study aimed at comparing the fracture resistance of trepanned zirconia crowns with and without artificial ageing to zirconia crowns without trepanation. The null hypothesis was rejected and a significant difference between group A (control) and group B as well as between group A and group C was found.

The measured fracture loads were well above 1100 N for all specimens from all groups. Human bite forces have been analyzed in various studies [26,27,28,29]. For voluntary maximum bite forces, the review by van der Bilt reported forces between 878 N ± 194 N and 306 N ± 42 N [28]. Forces used for chewing food are typically lower. Schindler for example reports about 120 N (wine gum), 50 N (carrot), and 20 N (boiled potato) in the first chewing cycle [30]. From that perspective, all measured fracture loads observed in this study seem sufficient for clinical use.

There are several possible explanations for the reduced fracture loads of the repaired groups. The surface treatment applied during trepanation likely triggers tetragonal to monoclinic phase transformation, which changes the phase composition, which is known to significantly affect the mechanical properties of zirconia-based ceramics [31]. Besides this, surface machining defects might lead to reduced strength, while suitable polishing techniques can also maintain or improve strength, as for example is analyzed in detail in the round-robin tests by Spintzyk et al. [32]. The interface between composite and 5Y-PSZ seems to be a relatively weak area, based on the observed crack patterns shown in Figure 6 and Figure 7. Furthermore, the different young’s modulus of the repaired area might change the stress distribution and might lead to a more unfavorable load distribution. This effect can however not be quantified here, as no finite element analyses were conducted. It might be of interest to address this in future work in more detail.

Besides the question, of whether full fracture plays a role, fissure formation between the composite and zirconia is of interest. If a fissure becomes too large, bacteria might migrate to the abutment tooth resulting in a caries risk. Based on the SEM data fissures were detected on the surface in the present study, which became larger after thermomechanical ageing. However, this does not imply that the fissures reached the abutment tooth. Within the resolution limit of the conducted XRM analyses, no penetrating fissures were detected. Possible fissures with a width below 7.65 µm cannot be excluded based on the available data.

Furthermore, the ageing applied in this study has a rather short water exposure compared to the crown’s lifetime (about 10 days during thermomechanical cycling). Other authors found no significant positive effect of MDP-based liners after 60 days in water [16] or found spontaneous failure between composite and Y-TZP during 150 days of water storage [33]. Garcia et al. [34] tested the shear bond strength (SBS) of resin cements to ultra-translucent zirconia (Y-PSZ), whereas Clearfil Ceramic Primer Plus (used in this study) was among the best luting agents after 1 year of water storage. An SBS for Clearfil Ceramic Primer Plus of (22.5 ± 5.1) MPa after 24 h was found, which degraded to (17.6 ± 4.5) MPa after 1 year of water storage [34].

The number of estimated chewing cycles per year differs in literature and between individuals. DeLong et al. [35] for example estimated 250.000 chewing cycles per year, whereas Aboushelib estimated 500.000 cycles per year [36]. Following Aboushelib the 1.000.000 cycles performed in this study represent about 2 years of function.

The results of the current study are affected by several limitations. The boundary conditions applied in with some mobility, rather rigid polyurethane support was chosen for the crowns, which was manufactured in a reproducible way. Furthermore, the load was introduced via a sphere as can be often seen in in vitro testing. The resulting contact areas differ from the clinical situation, where the contact situation varies strongly depending on the type of load (for example normal chewing or bruxism) and the antagonist situation. Especially in cases where the fracture occurred directly at a contact area/point, the chosen boundary condition will probably lead to different behavior in various clinical situations. While it was intended that one of the contacts was on the repaired area, this was not the case for all specimens, due to height deviations that resulted from the manual repair process. However, these deviations are not of paramount importance here, as the fracture loads are rather high in all tested circumstances.

In vitro repair of endodontic access cavities has also been performed by Scioscia et al. [37] for monolithic and veneered zirconia-based crowns. It can be concluded that endodontic access and adhesive restoration result in reduced fracture loads, whereas two-step filling with silica coating, silanization, and bonding of the marginal crown led to improved fracture loads. Marginal quality was addressed but limited to the near-surface region, as the analysis was SEM-based. Grobecker-Karl et al. [38] as well tested monolithic and veneered zirconia-based crowns and came to the conclusion that repaired monolithic restorations seem less susceptible to damage than veneered zirconia restorations. Schubert et al. [39] also tested repaired endodontic access cavities using 3Y-TZP and found no difference to their control group without access preparation. Mallya et al. [40] tested repaired Y-TZP and lithium disilicate crowns and found no significant difference in fracture tests.

Wiegand et al. [11] performed a clinical retrospective study of crowns that were repaired after endodontic access. 10-year survival was 48.8% and the authors came to the conclusion that ‘repairing endodontic access cavities with composite increases the longevity of single crowns and retainer restorations’ [11].

Future work should aim at further addressing the question of whether fissure formation occurs in thermomechanical aged specimens, for instance by performing bacteria penetration tests in a suitable setup.

## 5. Conclusions

Based on the findings of this study following the conclusions can be drawn:

- Trepanned and composite repaired 5Y-PSZ crowns show lower fracture loads than 5Y-PSZ crowns without trepanation.
- The fracture load of trepanned and repaired crowns after thermomechanical ageing representing about 2 years of function appears reasonably high (>1100 N).

## Data Availability

The data are available on reasonable request.
